# Chronic Nephropathy from Dietary Hyperoxaluria: Sustained Improvement of Renal Function after Dietary Intervention

**DOI:** 10.7759/cureus.1105

**Published:** 2017-03-20

**Authors:** Yijuan Sun, Bruce L Horowitz, Karen S Servilla, Joanna R Fair, Darlene Vigil, Kavitha Ganta, Larry Massie, Antonios H Tzamaloukas

**Affiliations:** 1 Epidemiology, Raymond G Murphy VA Medical Center; 2 Medicine, University of Utah School of Medicine, University of Utah; 3 Nephrology, Raymond G Murphy VA Medical Center; 4 Radiology, University of New Mexico School of Medicine; 5 Medicine Service, Raymond G Murphy VA Medical Center; 6 Pathology Service, Raymond G Murphy VA Medical Center; 7 University of New Mexico School of Medicine

**Keywords:** dietary hyperoxaluria, oxalate nephropathy, chronic kidney disease

## Abstract

A 56-year-old man with stable chronic kidney disease (CKD) for two years following a single episode of calcium oxalate urolithiasis developed progressive elevation of his serum creatinine concentration. Urinalysis revealed pyuria and white cell casts, a few red blood cells, minimal proteinuria, and no crystals. Urine culture was sterile. Gallium scintigraphy was consistent with interstitial nephritis. Proton pump inhibitor intake was discontinued, and a short course of oral corticosteroids was initiated. Percutaneous kidney biopsy, performed because of the continued deterioration of renal function to a minimum estimated glomerular filtration rate (eGFR) value of 15 mL/min per 1.73 m^2^ and persistent pyuria, revealed deposition of oxalate crystals in the tubules and interstitium, pronounced tubular changes, and interstitial nephritis and fibrosis. Urinary oxalate excretion was very high, in the range usually associated with primary hyperoxaluria. However, investigations for primary or enteric hyperoxaluria were negative. He reported a diet based on various nuts high in oxalate content. Estimated oxalate content in the diet was, for years, approximately four times higher than that in the average American diet. The institution of a diet low in oxalates resulted in the rapid normalization of urinary oxalate excretion and urinary sediment and in the slow, continuous improvement of renal function to near normal levels (eGFR 59 mL/min/1.73 m^2^) before his death from a brain malignancy 3.5 years later. The manifestations of nephropathy secondary to dietary hyperoxaluria, including the urine findings, can be indistinguishable from other types of interstitial nephritis. The diagnosis of dietary hyperoxaluria requires careful dietary history and a kidney biopsy. Identifying dietary hyperoxaluria as the cause of CKD is important because the decrease in dietary oxalate intake without any other measures can lead to sustained improvement in renal function.

## Introduction

Hyperoxaluria may cause urolithiasis, nephrocalcinosis, acute kidney injury (AKI), and chronic kidney disease (CKD). Oxalate excreted in the urine is derived from both endogenous production and gastrointestinal absorption. Increased endogenous production of oxalates is encountered in primary hyperoxaluria or after ingestion of large amounts of compounds that are metabolized to oxalates, such as ascorbic acid and ethylene glycol. Increased gastrointestinal absorption of oxalates are encountered in certain intestinal diseases and/or surgical interventions causing steatorrhea (intestinal hyperoxaluria) or after ingestion of foods with high oxalate content (dietary hyperoxaluria). A type of mild hyperoxaluria associated with urolithiasis is labeled idiopathic because its pathogenesis has not been defined conclusively. 

The degree of urinary oxalate excretion may provide clues about the etiology of hyperoxaluria. Oxalate excretion is typically very high in primary hyperoxaluria, varies with dietary oxalate intake in enteric hyperoxaluria, and is reportedly slightly higher than the normal range (< 45 mg/24-h) in mild and dietary hyperoxaluria. The following oxalate excretion rates were reported in one review [1]: A) Primary hyperoxaluria type 1, which is by far the most common variety of primary hyperoxaluria, > 90 mg/24-h; B) enteric hyperoxaluria > 90 mg/24-h; C) idiopathic hyperoxaluria < 63 mg/24-h; and D) dietary hyperoxaluria < 54 mg/24-h. The development of renal failure decreases urinary oxalate excretion and complicates the diagnosis and differentiation of hyperoxaluria. 

CKD from dietary hyperoxaluria is the topic of this report. The following set of criteria characterize a hyperoxaluric state as dietary hyperoxaluria: (a) documentation of the absence of primary, enteric, or idiopathic hyperoxaluria; (b) documentation of high dietary oxalate content; (c) documentation of hyperoxaluria during periods of high oxalate intake; and (d) normalization of urinary oxalate excretion after reduction in oxalate intake. We report a patient with advanced CKD who fulfilled all the criteria for the diagnosis of dietary hyperoxaluria, although he repeatedly exhibited oxaluria in the range of primary hyperoxaluria. This subject had prolonged follow-up with normalization of his renal function after cessation of high oxalate intake.

## Case presentation

A 56-year-old white male was evaluated for progressive CKD. He had a history of partial seizures treated with lamotrigine and levetiracetam. His other medications included clonazepam for anxiety and rabeprazole for symptomatic gastroesophageal reflux. He had a remote history of depression and alcoholism with binge drinking. At the age of 54 years, he developed left ureteral colic necessitating retrograde ureteral catheterization and laser lithotripsy of two small ureteral stones composed of calcium oxalate (70%) and apatite (30%). The serum creatinine concentration, which had been normal for 14 years until that point, was 2.1 mg/dL at the time of the renal colic. Ultrasonography and spiral computed tomography (CT) showed mild hydronephrosis of the left kidney but failed to show any other stones in the urinary tract at that time. He increased his fluid intake after that episode. 

**Figure 1:**
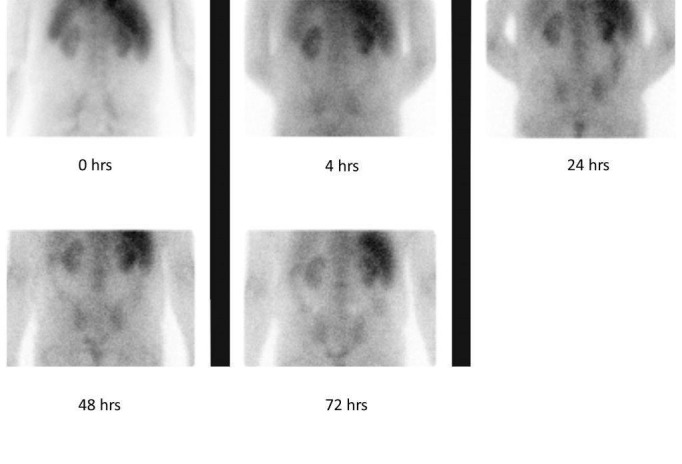
Gallium Scintigraphy The picture is consistent with interstitial nephritis. The kidneys retained the isotope even at 72 hours after its administration.

Two years later, serum creatinine concentration, which had remained in the 1.7 - 2.1 mg/dL range, rose progressively to 2.87 mg/dL in three months. Urinalysis revealed trace protein, a few red cells, numerous white cells, eosinophils (2%), white cell casts, and no bacteria or crystals. His urine culture was sterile. He refused a kidney biopsy initially. On repeated examinations, serum calcium and phosphorus levels were normal and the serum parathyroid hormone (PTH) level was consistently elevated while 25(OH)-cholecalciferol and 1, 25(OH)-cholecalciferol levels were consistently depressed. Serum complement (C3, C4) levels were normal. Serology was negative for hepatitis B and C antibodies, antinuclear antibodies (ANA), and antineutrophil cytoplasmic antibodies (ANCA). A serum angiotensin-converting enzyme (ACE) level and erythrocyte sedimentation rate (ESR) were also normal. Gallium scintigraphy suggested the diagnosis of interstitial nephritis (Figure 1). 

**Figure 2:**
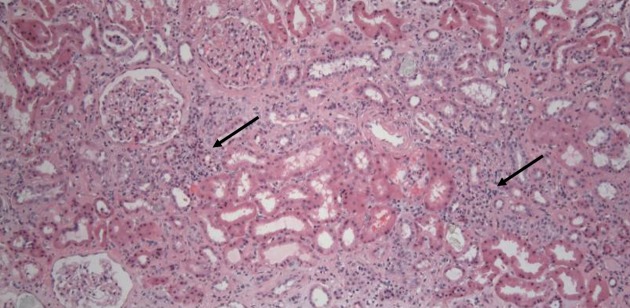
Renal Biopsy

Rabeprazole was discontinued and oral prednisone was started. However, urinalysis continued to show pyuria, white blood cell casts, and modest proteinuria, while the serum creatinine concentration continued to rise to a high of 4.19 mg/dL (estimated eGFR calculated by the modification of diet in renal disease (MDRD) formula - 15 mL/min per 1.73 m2 body surface area (BSA)). A percutaneous kidney biopsy was performed at that time. There was no glomerular pathology on histology. The tubules showed focal flattening of the epithelium and vacuolization of the epithelial cells. Approximately 60% of the biopsied sample showed tubular atrophy and interstitial fibrosis on a trichrome stain. Moderate arterial and arteriolar sclerosis were also noted. Focal dense infiltrates of mononuclear cells were seen in the interstitium (arrows in Figure 2).

**Figure 3:**
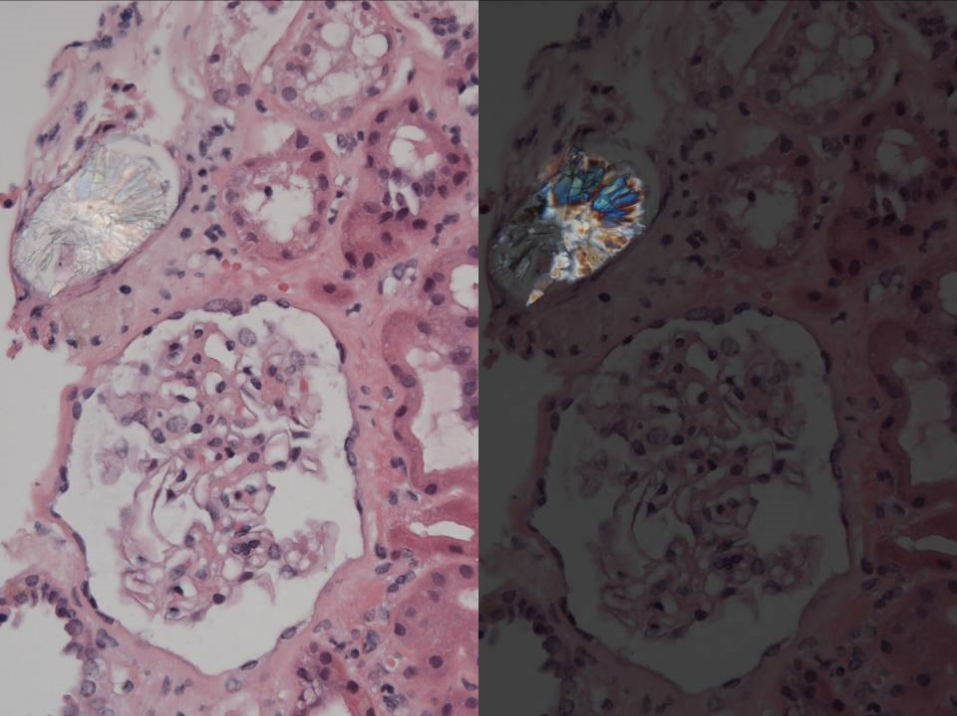
Renal Histology The picture shows oxalate deposits.

Numerous calcium oxalate crystals, which were birefringent under polarized light, and a few calcium phosphate crystals were seen in the lumen of many tubules (Figure 3).

Immunofluorescence showed segmental deposition of IgM and C3 in the mesangium, scattered fibrin deposits in the interstitium, and focal deposition of C3 in tubular basement membranes. Electron microscopy confirmed the prominent degenerative changes of the epithelium. The glomeruli showed only focal effacement of the interdigitating foot process. Electron-dense deposits were not found. The pathologic diagnosis was a hyperoxaluric state with chronic interstitial nephritis, widespread calcium oxalate deposition in the tubules, marked tubular distention, and signs of tubular injury.

Repeated spiral CT done without contrast showed numerous small stones in the kidneys. A 24-hr urine sample showed hyperoxaluria, hypocitraturia, and hypocalciuria. Screening for primary hyperoxaluria revealed the absence of any family history, renal stones, or CKD. Urinary excretion of glycolate - 7 µg/mg creatinine (normal range: < 79 µg/mg creatinine), undetectable glycerate (normal range: < 9.5 µg/mg creatinine), and glyoxylate - 1.2 µg/mg creatinine (normal range: < 3.0 µg/mg creatinine) were normal. On three occasions, serum ascorbic acid was 1.30, 0.22, and 1.21 mg/dL (normal range: 0.20 - 1.90 mg/dL). Investigation for enteric hyperoxaluria was also negative. There was no history of organic disease of the gastrointestinal tract, no previous surgical interventions in the abdomen, and on numerous occasions, liver function tests, serum albumin, calcium, phosphate, magnesium, electrolytes, and glucose concentrations were persistently normal. Detailed dietary history revealed that he had consumed almost exclusively nuts, including peanuts, walnuts, and nut butter for several years. The average daily oxalate content in his diet was estimated conservatively at 800 mg, a value which is a multiple of the reported average values for oxalate intake in the general American population [2].  

After repeated dietary consults, he drastically changed his diet. He reduced oxalate intake and increased water intake. Pyridoxine and Vitamin D3 preparations were added to his medications. Two weeks after the institution of a low oxalate diet, the pyuria and white cell casts disappeared and his serum creatinine concentration started decreasing slowly. His serum PTH and Vitamin D levels became normal. He developed a rapidly growing glioblastoma and expired 3.5 years after the kidney biopsy. The last serum creatinine level was 1.25 mg/dL (eGFR 59 mL/min per 1.73 m2 BSA). Repeated urine examinations consistently showed no sediment while proteinuria, which had reached a peak of 0.6 g/24-h soon after the change in diet, decreased to normal levels (< 0.2 g/g creatinine). 

Table 1 shows urinary excretion of oxalate and other relevant substances during the high and low oxalate intake periods. Twenty-four hour urine collections for determination of urine volume and of the rates of excretion of oxalate, creatinine, citrate, calcium, phosphorus, and uric acid were performed between one and four times in each of the two periods studied. In Table 1, the superscript 1 indicates two separate urine collections, the superscript 2 indicates four separate collections and the superscript 3 denotes one urine collection. Urinary oxalate excretion was consistently very high during the period of high oxalate intake and was within normal range after reduction of the oxalate intake. In addition, in one study during the high oxalate intake period, his urine pH was 5.42, ammonium was 28 mmol/24-h (normal range: 14-62 mmol/24-h), and sulfate was 12 mmol/24-h (normal range: < 30 mmol/24-h). 

**Table 1:** Urine Excretion Rates at High and Low Oxalate Intakes Serum creatinine concentration was 3.02 ± 0.13 mg/dL in the period of high oxalate intake and 2.34 ± 0.39 mg/dL during the early stages of low oxalate intake when the 24-hr urine collections took place. Superscript 1: Two urine collections. Superscript 2: Four urine collections. Superscript 3: One urine collection.

Variable	Normal Range	High Oxalate Intake Period	Low Oxalate Intake Period
Volume, L/24-h	1.0 - 2.0	3.7 ± 1.0^1^	5.1 ± 1.0^2^
Creatinine, mg/24-h	800 - 2,000	1,455 ± 485^1^	1,576 ± 340^2^
Oxalate, mg/24-h	< 45	238 ± 36^1^	36 ± 4^2^
Oxalate/creatinine, mg/g	1.6 - 37	228 ± 98^1^	36 ± 5^2^
Citrate, mg/24-h	> 320	84 ± 25^1^	105 ± 15^1^
Calcium, mg/24-h	< 250	21^3^	85 ± 35^1^
Phosphorus, mg/24-h	< 1,100	945^3^	1,120^3^
Uric acid, mg/24-h	< 700	213 ± 57^1^	389 ± 52^1^

## Discussion

This case raises two main points. The first point is that chronic intake of food items with a high content of oxalate may lead to hyperoxaluria with urinary oxalate excretion similar to the degree usually seen in primary hyperoxaluria. The component of urinary oxalate excretion that is derived from gastrointestinal absorption is determined by the oxalate content of the diet and the rate of absorption of oxalate in the intestines. Food items high in oxalate include leafy vegetables, such as spinach, various nuts, e.g., peanuts, and tropical fruits, including Averrhoa carambola (starfruit) and Averrhoa bilimbi. 

The average daily dietary oxalate intake content in the United States is 214 mg in men, 185 mg in older women, and 183 mg in younger women; spinach accounts for > 40% of the oxalate intake [2]. Factors leading to high rates of oxalate absorption from the gut include the presence of medical conditions or surgical interventions leading to steatorrhea, low dietary content in calcium and magnesium, both of which bind oxalate in the gastrointestinal tract and decrease its absorption and renal excretion, and the absence from the intestinal flora of certain species of bacteria, in particular, Oxalobacter formigenes, an anaerobic bacterium that metabolizes oxalate. The absence of this bacterium from the gut has been associated with hyperoxaluria. Administration of Oxalobacter formigenes preparations may be useful in hyperoxaluric states, including primary hyperoxaluria and enteric hyperoxaluria.

Although there is substantial variation between individuals, urinary oxalate excretion rises in parallel with dietary oxalate intake when other variables potentially affecting oxaluria are under control [3]. In addition to oxalate intake, intake of oxalate precursors may cause hyperoxaluria. Ascorbic acid, pyridoxylate, which is a combination of glyoxylic acid and pyridoxine, and hydroxyproline are potential sources of oxalate that have caused hyperoxaluric renal disease in clinical and experimental studies. Finally, hyperoxaluria and oxalate nephropathy may develop as a result of the oxalate salts of parenteral medications.

The principal manifestations of acute intoxication from ingestion of food items, drugs, or toxic substances with high oxalate content involve the gastrointestinal tract (pain, nausea, bloody vomiting, bloody diarrhea), the nervous system (tetany, manifestations of cerebral edema), and the kidneys (proteinuria, oligo-anuria). Surviving patients with AKI may recover normal renal function even if they develop severe renal failure requiring dialysis for some time [4-6]. 

CKD may develop as a consequence of dietary hyperoxaluria. The cause of hyperoxaluric CKD was consumption of peanuts in one patient [7] and “juicing” (chronic intake of large volumes of juices extracted from various vegetables) in three other patients [8]. Deposition of oxalate crystals in renal tubules, extensive degenerative changes in the tubules, and varying degrees of interstitial inflammation and fibrosis were the main histological characteristics of nephropathy in these case reports. Renal function improved after cessation of the high oxalate intake [7-8]. 

CKD from dietary hyperoxaluria has similar histological characteristics with all other types of oxalate nephropathy. Sterile pyuria with white cell casts is found in all types of interstitial nephritis. Interestingly, calcium oxalate crystals, which could direct the clinicians towards the diagnosis of oxalate nephropathy, were not seen in the urine of our patient or in several published reports of oxalate nephropathy.

Gallium scintigraphy has been used to distinguish between acute interstitial nephritis and other types of AKI. We found no reports of gallium scintigraphy in hyperoxaluric patients. In our patient, gallium scintigraphy was positive, supporting the initial clinical diagnosis of interstitial nephritis. Clinical picture, urinalysis, and imaging methods are usually not sufficient for differentiating between oxalate nephropathy and other types of interstitial nephritis. A kidney biopsy is required. Deposition of C3 and immunoglobulins in the tubular basement membrane has also been found in enteric hyperoxaluria [4]. 

New developments on molecular mechanisms of tubular epithelial damage and interstitial inflammation have shed light on the mechanism of oxalate nephropathy. Oxalate binding proteins in kidney tissues and mediators of attachment of calcium oxalate to tubular cells have been characterized. Hyperoxaluria causes up-regulation of Kidney Injury Molecule-1 (KIM-1). Urinary oxalate levels were found to correlate with tumor necrosis factor and Fas ligand levels and to be associated with apoptosis of the renal tubular cells. Inflammasomes are cytosolic high molecular weight complexes that are parts of the innate immune system. The nucleotide-binding domain, leucine-rich inflammasome (NALP3, or NLRP3, or cryopyrin), oligomerizes upon activation and recruits protease caspase 1 to form an inflammasome protein complex. The activated caspase 1 cleaves the inactive precursors of IL-1β and Il-18 leading to the generation of the active forms of these cytokines. Studies in experimental animals showed that hyperoxaluria activates the NALP3 inflammasome and that NALP3-mediated inflammation is necessary for the development of oxalate nephropathy CKD [9].

The second important point illustrated by this case report is that recognition of dietary hyperoxaluria and reduction of the oxalate intake can lead to improvement of the renal function, which may be sustained for years. Dietary hyperoxaluria offers an ideal substrate for the study of the effect of normalization of oxaluria on the course of hyperoxaluric CKD because the only cause of hyperoxaluria in our case was high dietary oxalate intake. Two previous reports [7-8] noticed an improvement in the renal function after normalization of oxalate intake. Our report indicates that, even when CKD has reached advanced stages, the improvement of renal function in dietary hyperoxaluria after a reduction of the oxalate intake can be sustained and significant, leading to normalization of both serum creatinine and urinary findings. Partial improvement of CKD has also been reported in a few patients with advanced hyperoxaluric CKD secondary to chronic intake of large amounts of ascorbic acid.

## Conclusions

Renal biopsy is needed for establishing the diagnosis of CKD secondary to dietary hyperoxaluria. In patients with CKD secondary to dietary hyperoxaluria, the institution of a diet low in oxalate leads to a decrease in urinary oxalate excretion and may lead to rapid normalization of the renal sediment and slow improvement of the renal function. Prevention of dietary hyperoxaluria, by avoiding excessive intake of foods high in oxalate or its precursors, should be a part of the dietary education of the public.
